# Altered Neurovascular Coupling Dynamics During Dobutamine‐Induced Acute Stress

**DOI:** 10.1002/brb3.71764

**Published:** 2026-09-15

**Authors:** Rui Wang, Pengling Ren, Xia Ma, Linkun Cai, Tingting Zhang, Fengxia Yu, Liang Zhu, Zhenghan Yang, Yawen Liu, Zhenchang Wang, Peng‐Gang Qiao

**Affiliations:** ^1^ Precision and Intelligence Medical Imaging Lab, Beijing Friendship Hospital Capital Medical University Beijing China; ^2^ Department of Radiology, Beijing Friendship Hospital Capital Medical University Beijing China; ^3^ Department of Ultrasound, Beijing Friendship Hospital Capital Medical University Beijing China; ^4^ Rehabilitation Hospital Affiliated to National Research Center for Rehabilitation Technical Aids Beijing China

**Keywords:** acute stress, cerebral blood flow, dobutamine, resting‐state fMRI, neurovascular coupling

## Abstract

**Background:**

Neurovascular coupling (NVC) is essential for regulating brain metabolism and energy supply and may be altered under acute stress. This study aimed to investigate how acute stress induced by dobutamine affects NVC.

**Materials and methods:**

This study enrolled 38 healthy participants undergoing a resting‐state fMRI scan and an arterial spin labeling MRI scan prior to and during a stress test induced by dobutamine. Amplitude of low‐frequency fluctuation (ALFF), regional homogeneity (ReHo), and cerebral blood flow (CBF) were calculated. Acrossvoxel CBF‐ReHo and CBF‐ALFF correlations were analyzed to evaluate the NVC within the whole gray matter, and the regional NVC of the brain was assessed with the CBF/ReHo and CBF/ALFF ratios.

**Results:**

The blood pressures at the ventricle systolic state (SBP) and diastolic state (DBP) were significantly increased after the infusion of dobutamine. The CBF/ReHo ratios in the postcentral cortex and the middle cingulate cortex under stress were significantly higher and lower, respectively, than the CBF/ReHo ratios in the postcentral cortex and the middle cingulate cortex under the resting state. Logistic regression analysis showed that DBP in the resting state (odds ratio [OR] 0.817, 95% confidence interval [CI] 0.692–0.965, *p =* 0.017) and SBP in the stress state (OR 1.218, 95% CI 1.067–1.390, *p =* 0.004) were significantly related to the increase in CBF/ReHo ratios in the left postcentral cortex.

**Conclusion:**

Dobutamine‐induced acute stress modulated NVC dynamics. Participants with lower DBP in the resting state and higher SBP during acute stress were more prone to have increased NVC induced by stress.

**Relevance Statement:**

Acute cardiovascular stress induced by dobutamine altered neurovascular coupling dynamics, and individuals with lower resting diastolic pressure and greater systolic responses showed stronger stress‐related NVC enhancement.

**Abbreviations**
ALFF: Amplitude of low‐frequency fluctuation
ASL: Arterial spin labeling
CBF: Cerebral blood flow
CSF: Cerebrospinal fluid
DBP: Diastolic state
GM: Gray matter
ICA: Internal carotid artery
MAP: Mean arterial pressure
MRA: Magnetic resonance angiography
NVC: Neurovascular coupling
OR: Odds ratio
ReHo: Regional homogeneity
SBP: Systolic state

## 1 Introduction

Despite a thorough understanding of the endocrinology and pathophysiology responses to acute stress, it remains an independent risk factor for cardio‐cerebrovascular events, both in individuals with pre‐existing cardiac conditions and in those without known cardiovascular or neurological disease (Kivimäki et al. 2023; Agorastos and Chrousos 2022; Yoganathan et al. 2023; Poller et al. 2022; Tian et al. 2022; Vaccarino et al. 2018; Osborne et al. 2020). This observation has prompted growing interest in the brain–heart axis, an integrative framework that captures the bidirectional communication between central and peripheral systems and its role in stressrelated vulnerability (Tian et al. 2022; Osei et al. 2024; Vaccarino et al. 2021; Weissman and Mendes 2021). The brain and heart are interconnected through a complex network of physiological, neurohumoral, and immunological pathways that continuously regulate autonomic, vascular, and metabolic homeostasis (Mohanta et al. 2023; Rossi et al. 2022). Central to this interaction is neurovascular coupling (NVC), the fundamental physiological mechanism that precisely matches local neuronal activity with a corresponding change in regional cerebral blood flow (CBF) (Iadecola 2017; Tournissac et al. 2024; Han et al. 2019; Han et al. 2020; Archila‐Meléndez et al. 2020). Traditionally, investigations of NVC have focused on localized neural activation and cerebrovascular responses within the brain (Iadecola 2017; Han et al. 2020; Guo et al. 2019; Hu et al. 2019; Li et al. 2021). However, emerging evidence suggests that NVC extends beyond purely neural domains, reflecting systemic physiological interactions with the cardiovascular system (Aires et al. 2020; Mohanta et al. 2022; Moazzami et al. 2020; Walsh et al. 2024; Alan et al. 2026). These findings position NVC as a promising lens through which to explore the functional connectivity between the brain and heart.

Nevertheless, the modulation of NVC under acute stress remains largely unexplored (Jammal Salameh et al. 2024; Gargadennec et al. 2022; Mathew et al. 2021; Zhao et al. 2023). Current studies have primarily examined either cardiovascular or cerebral alterations in isolation, providing limited insight into how acute stress influences the integrated brain–heart network via NVC (Vaccarino et al. 2021; Mikail et al. 2024; Dai et al. 2023; Bai et al. 2022; Kim et al. 2023). This knowledge gap is partly due to the technical and ethical challenges of imaging stress‐related cerebrovascular responses in healthy individuals. Moreover, NVC is shaped by multiple demographic and physiological factors, including sex, blood pressure, and age, which further complicate its assessment (Tawakol et al. 2019; Rosovsky et al. 2024; Haider et al. 2021).

Given these limitations, there is a pressing need for experimental models that can safely and reproducibly induce acute stress while allowing simultaneous assessment of cardiovascular and cerebrovascular function. Dobutamine, a synthetic β‐adrenergic receptor agonist, offers a controlled and well‐characterized means to elicit sympathetic activation, mimicking the “fight‐or‐flight” response observed during acute psychological or physiological stress. By transiently elevating heart rate, myocardial contractility, and systemic blood pressure, dobutamine provides an effective experimental paradigm to examine how peripheral cardiovascular reactivity modulates cerebral perfusion and neurovascular coupling (NVC). Such pharmacologically induced hemodynamic perturbations are expected to alter CBF dynamics and vascular tone, thereby influencing NVC patterns within stress‑responsive cortical regions. Clarifying this interaction is essential for understanding how sympathetic cardiovascular activation translates into cerebral hemodynamic adjustments and how these mechanisms might contribute to stress‑related vulnerability of the brain–heart axis.

In this context, the present study was designed to characterize neurovascular coupling dynamics under dobutamine‐induced acute stress. To operationalize NVC, we adopted a multimodal neuroimaging approach, quantifying it as the ratio of regional CBF to indices of spontaneous neuronal activity, specifically Regional Homogeneity (ReHo) and the amplitude of low‐frequency fluctuation (ALFF). This CBF/ReHo (or CBF/ALFF) ratio serves as a quantitative biomarker reflecting the efficiency of blood flow supply relative to local neural demand. Using this framework, our study aims to reveal how stress‐related autonomic and hemodynamic changes modulate these NVC indices at both systemic and cerebral levels. By integrating vascular reactivity, systemic physiological parameters, and these specific neuroimaging biomarkers, we seek to offer novel insights into the brain–heart connection under sympathetic stress.

## 2 Materials and Methods

### 2.1 Participants

Totally, 52 healthy volunteers were recruited through advertising. All volunteers were non‐smokers and had no recorded neurological disorders or history of consciousness loss due to head injuries. During screening, detailed medical history inquiries and resting electrocardiogram (ECG) monitoring were performed to exclude cardiovascular disease, arrhythmia, or any abnormal cardiological findings that could increase risk under dobutamine challenge. Individuals with ocular or vascular diseases, psychiatric disorders, or systemic conditions potentially affecting cerebral perfusion or MRI eligibility were also excluded. Forty‑eight participants who passed psychological and cardiological screening were enrolled. All participants refrained from cigarette smoking, tea, coffee, β‑blockers, and anti‑anginal medication for at least 24 h before MRI. This research was conducted as part of a multi‐investigator study, which required all participants to undergo additional experimental procedures. Finally, data of 38 participants were analyzed in view of data's integrity. Before the study, the informed consent forms were signed by all participants, and the study protocol was approved by the Hospital Research Ethics Committee (2020‐P2‐155).

### 2.2 Study Design and Procedure of Acute Stress Test

**FIGURE 1:**
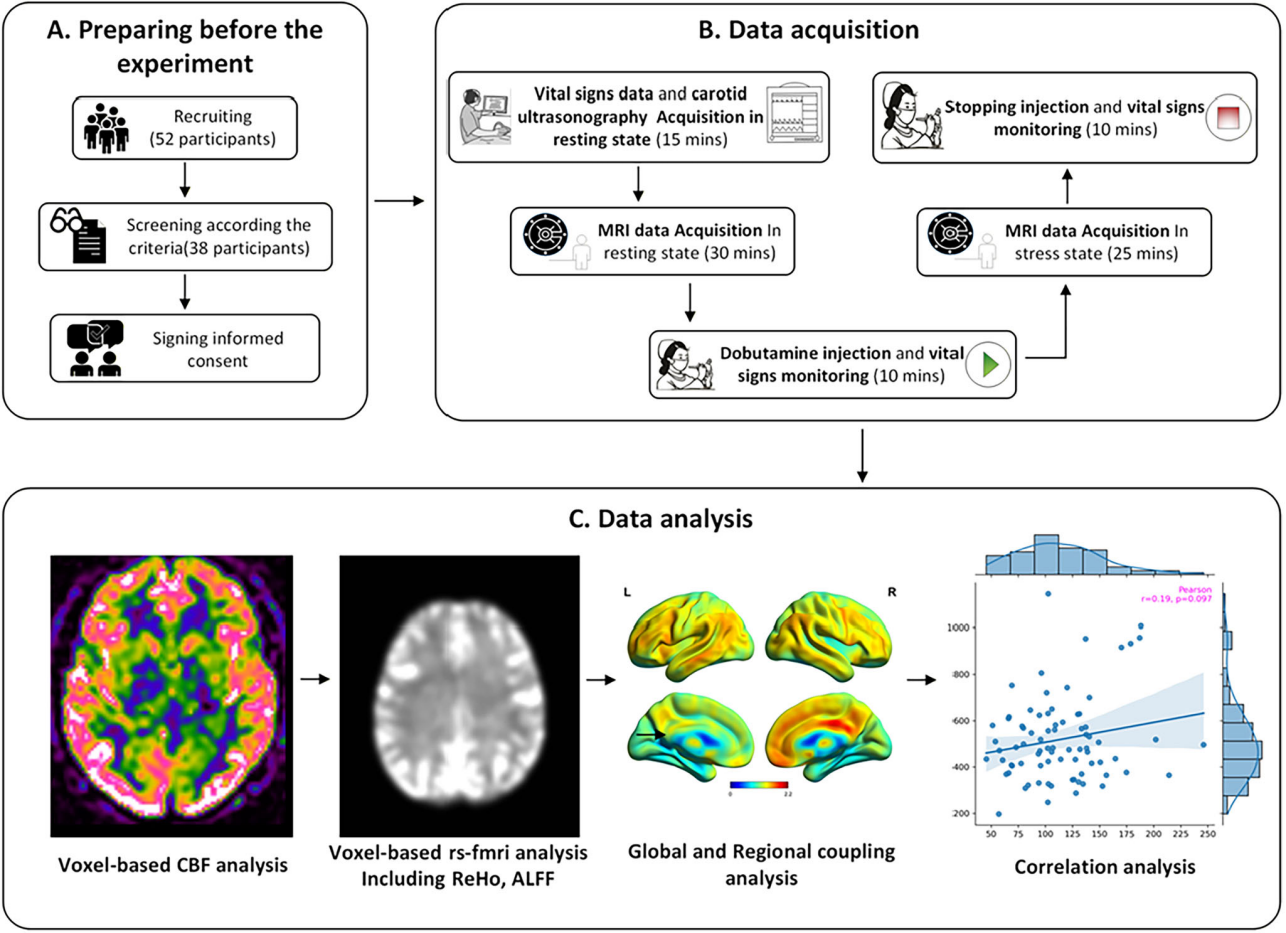
Schematic overview of the experimental design and data analysis. (A) flowchart of the participant enrollment; (B) flowchart of the experiment; (C) data acquisition and analysis procedure. CBF, cerebral blood flow; rs‐fMRI, resting‐state functional magnetic resonance imaging; ReHo, regional homogeneity; ALFF, amplitude of low frequency fluctuations.

The study was designed to be self‐controlled to explore the effects of acute stress produced by dobutamine on NVC. Figure 1 shows the flowchart of the study and procedures of data processing. The MRI was acquired with the standard procedures under resting state, and thereafter dobutamine was intravenously infused (dose: 5–20 µg/kg/min). During the dobutamine infusion, electrocardiogram, heart rate, blood pressure, and oxygen of participants were continuously monitored by the portable Multi‐parameter Vital Signs Monitor (BeneVision, Shenzhen Mindray Bio‐medical Electronic Co. Ltd, Shenzhen, China). The previous study indicated that the effect of infusion of dobutamine reached a plateau at 2 min and could be maintained at a steady state by continuous infusion (Ogoh et al. 2017). Once the physiological state of the participants reached steady state at the arterial pressure endpoint, which was defined as a change of less than 5 mm Hg in mean arterial pressure (MAP) over a duration of 2 min, MR scanning under stress state was conducted. After completion of the MR scanning, the dose of dobutamine was gradually decreased, and the physiological indices were monitored simultaneously until heart rate and blood pressure/oxygen were stable. The subsequent sections provided a description of the data acquisition and analysis details.

### 2.3 Ultrasound Data Acquisition

**FIGURE 2:**
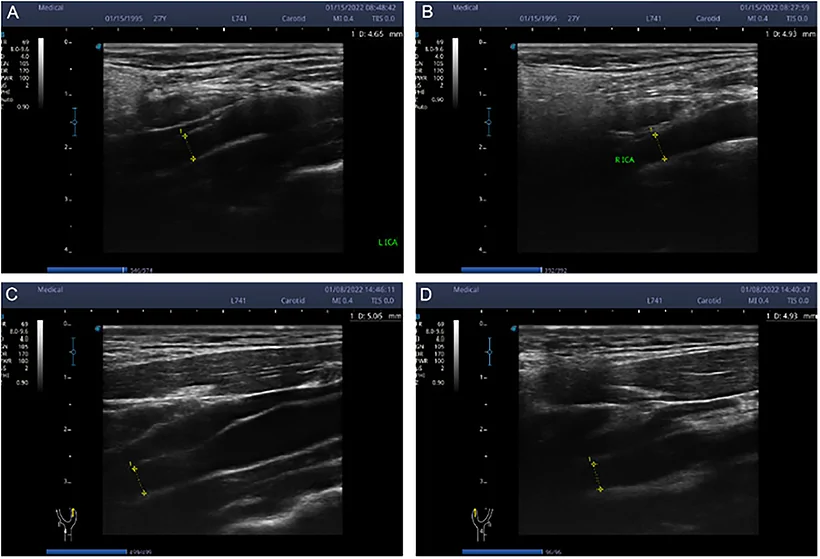
The ultrasonic images of two participants to show diameter measurement for the internal carotid artery (ICA) using carotid ultrasonography. (A‐B) the diameters of the left ICA and right ICA of participant 1; (C‐D) the diameters of the left ICA and right ICA of participant 2.

Once the participants' physiological state was stabilized, an ultrasonic scan was conducted by one operator using a color ultrasonic diagnosis system (Resona 7, Mindray, Shenzhen, China) equipped with a 3–9 MHz linear transducer. The diameters of bilateral internal carotid arteries (ICAs) were measured at 1.0–1.5 cm distally away from the carotid bifurcation by B‐mode images (Figure 2).

### 2.4 Acquisition of MRI Data

All participants were conducted with a set of high‐resolution T1‐weighted imaging, 3D pseudocontinuous arterial spin labeling (ASL) scanning, rs‐fMRI scanning, 3D time‐of‐light (3D‐TOF) magnetic resonance angiography (MRA) under resting state, and a set of 3D pseudocontinuous ASL scanning and rs‐fMRI scanning under stress state by using an MRI scanner (Ingenia 3.0‐T; Philips Healthcare, Best, Netherlands) equipped with a 16‐channel phased‐array head coil. A Foam padding was used to reduce the head motion, and earplugs were used to reduce the scanner noise for all participants.

The sequencing parameters of ASL were 36 axial slices with A slice thickness of 4 mm and A post‐label delay of 1500 ms, A TR/TE of 4852/10.7 ms, a reconstruction matrix of 128 × 128, a field view of 240 × 240 mm, and an excitations number of 3. The acquisition protocol of rs‐fMRI was conducted with: 30 slices with 4 mm of slice thickness, 3000 ms of TR, 35 ms of TE, 240 mm × 240 mm of FOV, and 64 × 64 of Matrix, with a total acquisition time of 10 min 9 s. The participants were asked with instruction to keep awake and their eyes closed and refrained from focusing on any specific thoughts.

### 2.5 CBF Data Preprocessing

The normalization of CBF maps to Montreal Neurologic Institute (MNI) space was implemented using the Statistical Parametric Mapping software (SPM8, https://www.fil.ion.ucl.ac.uk/spm) with MATLAB (Version R2014a, MathWorks, Natick, MA, United States) in the following consequential steps: (1) The raw CBF images of each participant were co‐registered to corresponding structural T1‐weighted images, which were then spatially normalized to MNI space with the deformation fields produced during the procedures of segmentation and normalization; (2) the transformed CBF images in MNI space were resampled to 3 × 3 × 3 mm^3^; (3) the individual variations in global perfusion of normalized CBF images, which were more sensitive to small changes in regional perfusion, were corrected by scaling each voxel in the CBF map with the mean of whole‐brain CBF; and (4) the standardized CBF maps were subjected to spatial smoothing with a 4 × 4 × 4 mm^3^ full‐width at half maximum (FWHM) of Gaussian kernel for further analysis.

### 2.6 Rs‐fMRI Data Preprocessing

The preprocessing of BOLD‐fMRI images was performed with SPM8 software and the Data Processing and Analysis for Brain Imaging (www.rfmri.org/dpabi) implemented in MATLAB. In each run, the initial 10 time points were excluded to address the equilibrium of signal and the adjustment of participants to the conditions. After correcting for time, the remaining functional photographs were initially made to the first volume to account for the motion of the head, which, in any participant, was equal to or less than 1.5 mm of movement or 1 degree of rotation in any angle. Subsequently, the functional pictures were subjected to spatial standardization to the standard template of MNI space. In order to further attenuate the influence derived from body motion and nonneuronal BOLD fluctuations, these changes caused by the motion of the head and the signals of cerebrospinal fluid (CSF) and white matter were considered as nuisance covariates and removed. Linear detrending and low temporal band‐pass filtering (0.01–0.08 Hz) were conducted in order to lessen the impact of very low frequency physiological noise. Finally, 3‐mm cubic voxels were used to resample the functional images for further analysis.

### 2.7 Calculation of Regional Homogeneity (ReHo) and ALFF

Regional homogeneity (ReHo) describes how different neighbors of the voxels are synchronized in terms of signal fluctuations. An individual ReHo map was created with the calculation of the temporal Kendall's coefficient concordance (KCC, also known as the ReHo value) of a specific voxel in relation to those of its 26 nearest neighbors, based on voxel‐wise (Zang et al. 2004). The ReHo maps were produced for each participant and subsequently processed by smoothing with a 6‐mm FWHM kernel. The ReHo calculations were performed using DPARSFA (http://rfmri.org/DPARSF).

As an R‐fMRI measurement, the ALFF is utilized to assess the fluctuations of spatial intensity of the BOLD signals reflecting the neural activities in specific brain areas and physiological/pathological conditions (Zang et al. 2007). The analysis of ALFF was performed using the toolbox DPABI (www.rfmri.org/dpabi). The time series for each voxel were filtered and converted into a frequency domain using the Fast Fourier Transform to produce the power spectrum. The ALFF for each voxel was calculated by taking the square root of the signal within the range of 0.01–0.08 Hz. To standardize the data and minimize the impact of individual ALFF, the ALFF of each voxel was divided by the global mean of ALFF values for each participant with the default mask from DPABI, excluding the signals of background and other non‐brain tissue, to create a standard whole‐brain ALFF map.

### 2.8 Across‐Voxel CBF–ReHo and CBF‐ALFF Coupling Analysis

Correlation analysis between CBF and ReHo and between CBF and ALFF across voxels for each participant was conducted to quantitatively evaluate the coupling within the gray matter (GM). To account for spatial autocorrelation among voxels introduced by spatial preprocessing and spatial smoothing, the effective degrees of freedom (df_eff_) were estimated according to the method proposed by Liang et al. (2013).

(1) $$d\;f_{eff}=\frac{V}{FWHM_{x}\times FWHM_{y}\times FWHM_{z}}\;-2$$

where V denotes the total number of voxels within the GM mask, and $\mathrm{FWH}M_{x}$, $\mathrm{FWH}M_{y}$, $\mathrm{FWH}M_{z}$ represent the spatial smoothness along the three cardinal axes.

For each participant, individual Pearson correlation coefficients (r) were computed and subsequently transformed into normalized z‐scores via Fisher's z‐transformation (z=arctanh(r)). To control for within‐subject clustering, second‐level group statistical analyses were conducted using individual z‐scores as independent observations (N=38). Normality and variance homogeneity of the z‐scores were verified before applying paired t‐tests (or non‐parametric Wilcoxon signed‐rank tests if normality was violated) to compare coupling strengths between the resting and stress states.

### 2.9 Voxel‐Wise Analysis of CBF, ReHo, ALFF, CBF/ ReHo Ratio, and CBF/ALFF Ratio

The voxel‐wise comparison was conducted to detect changes in CBF, ReHo, and ALFF. To quantify the amount of CBF or energy metabolism per unit of neural activity, the regional ratios of CBF/ReHo and CBF/ALFF were calculated on a voxel‐wise basis and then divided by the global mean value to enhance the normality. A sphere with a radius of 6 mm was centered around the maximal voxel within the clusters exhibiting significantly different CBF/ReHo ratio and CBF/ALFF ratio under different states for each participant to extract the values of CBF/ReHo and CBF/ALFF ratios for the following correlation analysis. These ratios are calculated as indirect indices of neurovascular coupling. This approach is based on the rationale that, under physiological conditions, local CBF is tightly coupled to neural activity. A higher ratio indicates a relative excess of perfusion relative to neural activity, while a lower ratio indicates a relative deficit. This method has been previously adopted in studies examining NVC under various conditions (Yu et al. 2019; Owens et al. 2024).

### 2.10 Correlation Analysis Between Regional NVC and Physiological Variables

The correlation between the CBF/ReHo ratio and physiological variables was evaluated with Pearson correlation analysis. As mentioned in the previous section, a 6‐mm‐radius sphere within clusters having significant CBF/ReHo ratios was used to extract the CBF/ReHo ratio. In order to determine the association between the changes of NVC induced by acute stress and the physiological variables, the CBF/ReHo ratios in brain areas with significant changes and physiological variables under the resting and stress states were subtracted. Next, the differential CBF/ReHo ratio and physiological variables were divided by the corresponding CBF/ReHo ratio under the resting state to acquire the percentage changes. Lastly, Pearson's correlation analysis was performed to evaluate the associations between the CBF/ReHo ratio change and physiological variables.

### 2.11 Statistics

#### 2.11.1 Demographic Data

The demographic data were analyzed by using SPSS 20.0 (Chicago, IL, United States), and Wilcoxon's signed‐rank test was performed to evaluate the differences in clinical characteristics between resting and stress states. A *p* < 0.05 was set as the level of significant difference.

#### 2.11.2 Voxel‐Based Intergroup Comparison in CBF, ReHo, ALFF, CBF/ReHo Ratio Map, and CBF/ALFF Ratio Map

In order to evaluate the local differences in the CBF, ReHo, ALFF, CBF/ReHo ratio, and CBF/ALFF ratio under different states, comparisons of voxel‐wise paired *t*‐tests were conducted with correction by a Gaussian random field (GRF) through combining voxel‐level *p* < 0.001, cluster‐level *p* < 0.05, and cluster size >100 for multiple comparisons, using the DPABI software V5.0 (http://rfmri.org/DPABI).

#### 2.11.3 Pearson's Correlation Analysis

SPSS (Version 20.0) was used for statistical analyses. The CBF‐ReHo and CBF‐ALFF correlation coefficients of the two time points were tested for normality using the Kolmogorov–Smirnov test; the data with normal distribution were analyzed with the paired *t*‐test, and data with non‐normal distribution were analyzed with the Mann–Whitney *U*‐test. The mean values of each cluster with significant inter‐group differences in CBF/ReHo ratio or CBF/ALFF ratio were extracted and correlated with physiological variables using Pearson correlation analysis. *p* < 0.05 was set as the level for significant difference.

#### 2.11.4 Logistic Regression Analysis

The variables used for regression analysis included age, heart rate (HR), respiration rate (RR), SBP, DBP, integrity of the Willis circle, ICA diameter, and BA diameter. A binary logistic regression model was established by introducing the binary dependent variables; these differences in CBF/ReHo ratio, which were greater than the median of the differences in CBF/ReHo ratio, were labeled as 1, and otherwise, labeled as 0. Regression analysis was conducted to identify the predictors of NVC under acute stress induced by dobutamine, using SPSS 20.0, and *p* < 0.05 was set as the level for significant difference.

## 3 Results

### 3.1 Physiological Features Under Different States

Thirty‐eight healthy participants were finally included in the study. Table 1 presented the physiological variables of participants under resting state and stress state. There were significant increases in HR (68.00 (59.25∼75.00) versus 84.50 (70.00∼101.25), *p* < 0.001), SBP (116.50 (112.50∼129.25) versus 161.50 (151.75∼166.75), *p* < 0.001), and DBP (73.00 (68.75∼80.00) versus 83.50 (80.00∼88.50), *p* < 0.001) between resting state and stress state.

**TABLE 1:** Demographic and physiological features of participants.

Characteristics	Resting state	Stress state	*p‐*value
Number of participants	38		—
Age (years)	26.00 (24.00–29.00)		—
Heart rate, BPM	68.00 (59.75–75.25)	84.50 (70.00–101.25)	< 0.001
Respiration rate, RPM	16.50 (15.00–20.00)	18.00 (15.00–22.00)	0.018
BA diameter, mm	3.00 (2.65∼3.45)		—
ICA diameter, mm			—
L_ICA	4.70 (4.36–5.07)		—
R_ICA	4.80 (4.31–5.05)		—
Blood pressure			—
SBP, mm Hg	116.50 (112.50–129.25)	161.50 (151.75–166.75)	< 0.001
DBP, mm Hg	73.00 (68.75–80.00)	83.50 (80.00–88.50)	< 0.001
Blood oxygen, %	99.00 (98.00∼100.00)	99.00 (98.00∼100.00)	> 0.05

*Note*: Data were presented by median (lower quartile, upper quartile).

Abbreviations: BA, basilar artery; BPM, beat per minute; DBP, diastolic blood pressure; ICA, internal carotid artery; L, left; R, right; SBP, systolic blood pressure.

### 3.2 Spatial Distributions of CBF, ReHo, ALFF, CBF/ReHo Ratio, and CBF/ALFF Ratio

**FIGURE 3:**
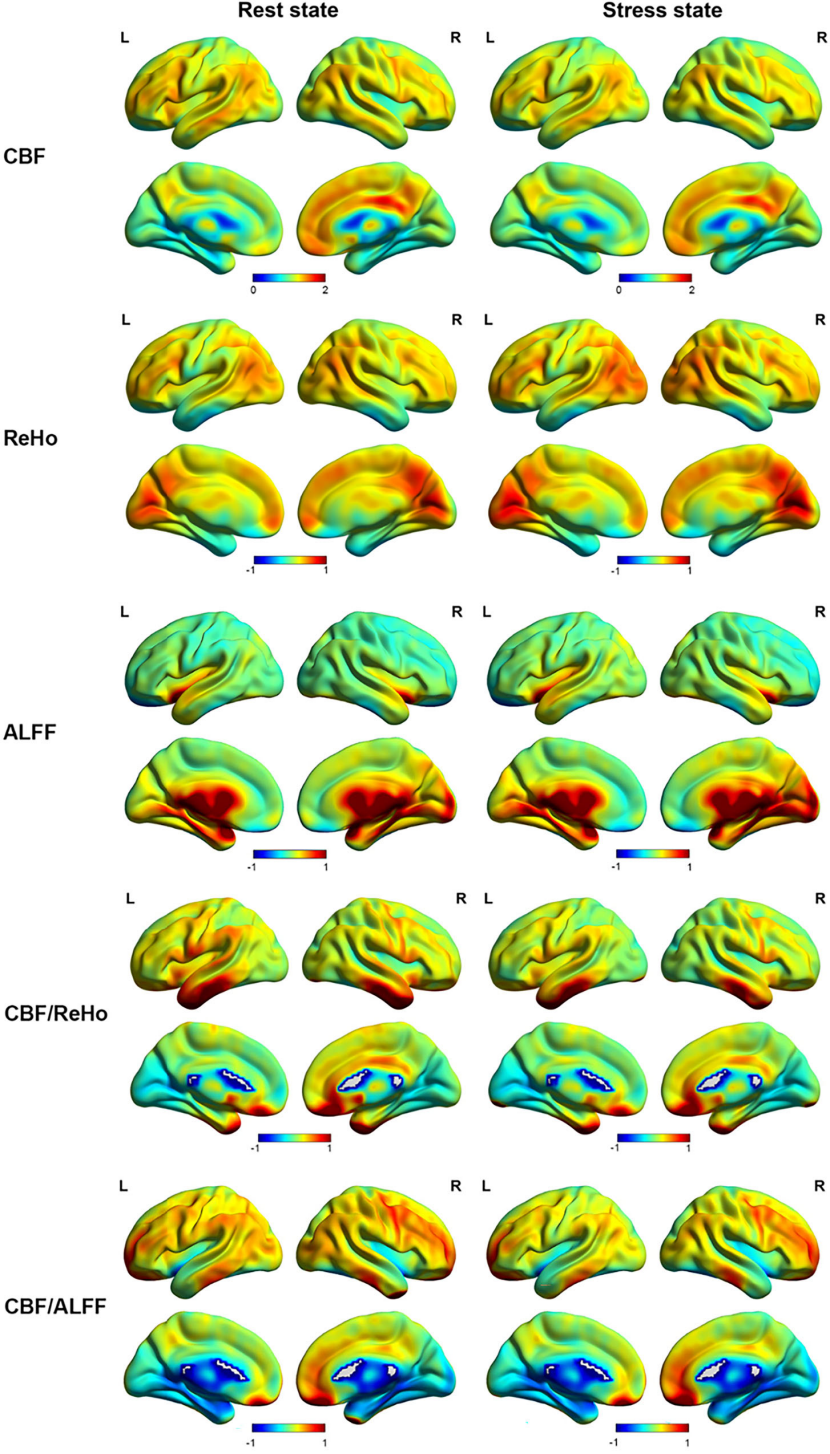
Spatial distribution of averaged maps. Left panel, spatial distribution in rest state. Right panel, spatial distribution in stress state. ALFF, amplitude of low‐frequency fluctuation; ReHo, regional homogeneity; CBF, cerebral blood flow; L: left; R: right.

As shown in Figure 3, the group‐averaged spatial distributions of CBF, ReHo, ALFF, and the derived coupling ratios (CBF/ReHo and CBF/ALFF) are shown separately for rest and dobutamine‐induced stress. Under the stress state, low CBF was mainly distributed over the superior and medial temporal cortex, postcentral cortex, middle cingulate cortex, insula cortex, and medial frontal cortex. Low ReHo and high ALFF values were mainly detected in the caudate cortex and postcentral cortex, respectively. High CBF/ReHo ratios were detected in the postcentral cortex, whereas low CBF/ReHo ratios were detected in the middle cingulate cortex. High CBF/ALFF ratios were mainly detected in the postcentral cortex and superior frontal cortex.

### 3.3 Changes of CBF‐ReHo and CBF‐ALFF Coupling in the Whole Gray Matter

**FIGURE 4:**
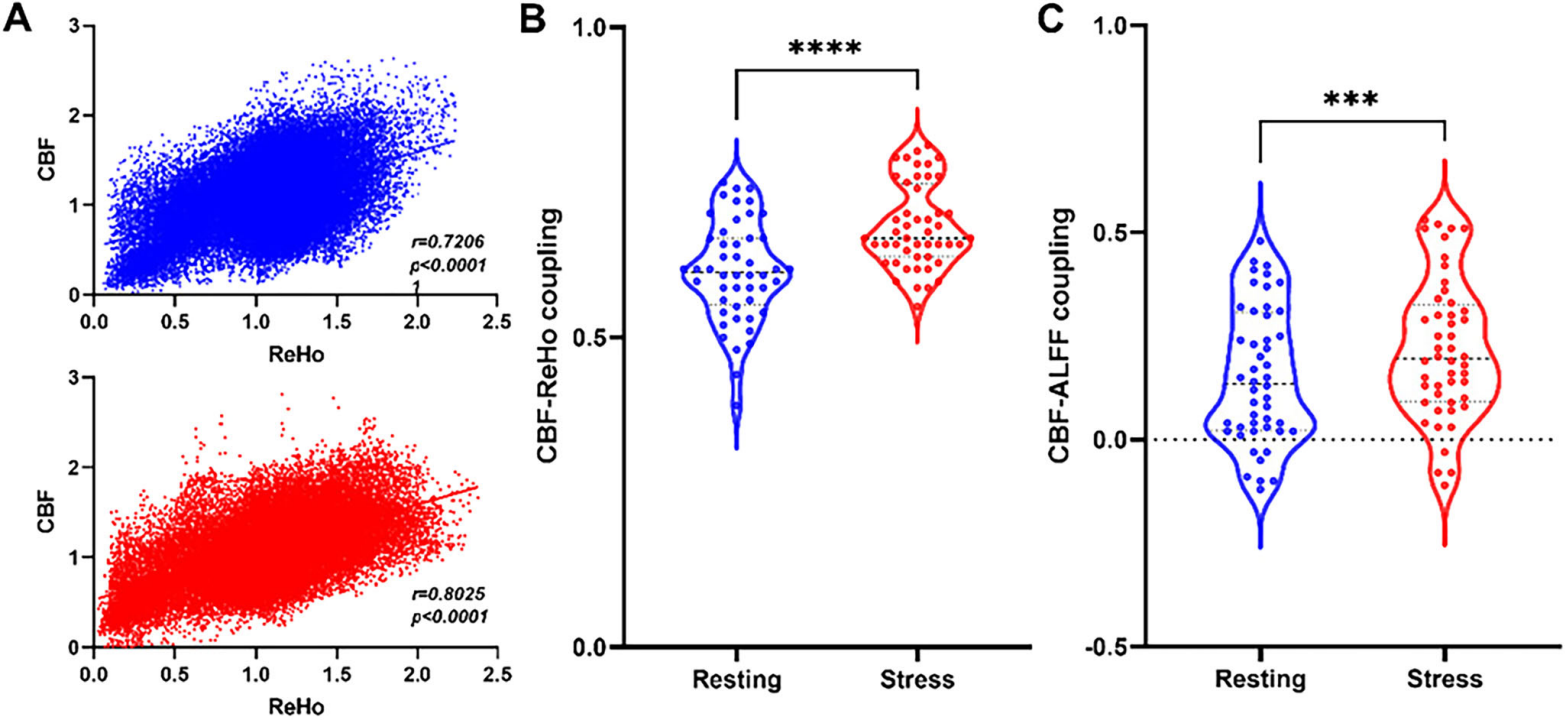
The changes of coupling between CBF‐ReHo and CBF‐ALFF under resting and stress states. (A) Scatter plots showing the spatial correlations across voxels between CBF and ReHo in the same participant under resting state (blue) and stress state (red). (B‐C) CBF‐ReHo and CBF‐ALFF within gray matter showed increased coupling under stress state when compared to resting state (*p* <0.001, *p =* 0.004 for CBF‐ReHo and CBF‐ALFF coupling, respectively). ALFF, amplitude of low‐frequency fluctuation; ReHo, regional homogeneity; CBF, cerebral blood flow; L: left; R: right.

All participants demonstrated significant spatial cross‐voxel correlations between CBF and ReHo, as well as between CBF and ALFF. Figure 4A shows two representative correlation maps illustrating the correlation between CBF and ReHo, one from each time point. Significant correlation was observed between CBF and ReHo at these two points; however, the CBF and ReHo coupling within the GM mask under the stress state was significantly greater when compared to the resting state (*p* < 0.001, Mann–Whitney *U*‐test; Figure 4B). As shown in Figure 4C, similar increased coupling between CBF and ALFF was detected under acute stress when compared to the resting state (*p =* 0.004; Mann–Whitney *U*‐test).

### 3.4 Changes in CBF, ALFF, ReHo, CBF/ReHo Ratio, and CBF/ALFF Ratio

When compared to the rest state, the CBF under the stress state showed no significant difference in the brain region of the posterior circulation but showed a significant decrease in the brain region of the anterior circulation, including the superior and medial temporal cortex, postcentral cortex, middle cingulate cortex, insula cortex, and medial frontal cortex (GRF correction, voxel level *p* < 0.001, pixel level *p* < 0.05; cluster size > 100) (Table 2). There was lower ReHo and higher ALFF in the caudate cortex and postcentral cortex, respectively, under the stress state when compared to the resting state (GRF corrected: *p* < 0.05, Table 2).

**TABLE 2:** Brain region with altered CBF, ReHo, and ALFF after dobutamine infusions.

Parameters	Regions	Cluster size (voxels)	Peak *t*‐values	MNI coordinates		
				*x*	*y*	*z*
CBF (Stress<Pre)	Temporal_Mid_R	2209	−7.9617	62	−6	−10
	Insula_L	751	−5.8297	−34	−6	−8
	Temporal_Sup_L	174	−5.8186	−60	−28	18
	Temporal_Mid_L	173	−5.5492	−60	−40	10
	Postcentral_L	235	−5.6733	−50	−24	28
	Postcentral_R	141	−5.7472	54	−14	52
	Frontal_Mid_R	121	−5.4284	34	44	40
	Cingulum_Mid_L	399	−6.3999	2	−6	36
ReHo (Stress<Pre)	Caudate_L	258	−4.9354	−12	4	16
	Caudate_R	88	−4.3295	22	12	18
ALFF (Stress>Pre)	Temporal_Mid_R	198	4.2633	58	−4	−34
	Temporal_Mid_L	308	4.3267	−56	−2	−22
	Temporal_Sup_R	432	4.4967	66	−18	−2
	Postcentral_L	1540	4.8233	−66	−4	22
	Postcentral_R	256	4.0039	58	−6	28

Abbreviations: ALFF, amplitude of low frequency fluctuations; CBF, cerebral blood flow; L, left; MNI, Montreal Neurological Institute; R, right; ReHo, regional homogeneity.

Under the stress state, the CBF/ReHo ratio displayed a significant increase in the left postcentral cortex and a decrease in the right middle cingulate cortex when compared to the resting state (GRF correction, *p* < 0.05; Table 3). CBF/ALFF ratios were significantly increased under stress state in the postcentral cortex and superior frontal cortex when compared to resting state (GRF correction, *p *< 0.05; Table 4).

**TABLE 3:** Brain region with altered CBF/ReHo after dobutamine infusions.

Regions	Cluster size (voxels)	Peak *t* values	MNI coordinates		
			*x*	*y*	*z*
Postcentral_L	6500	24.4105	48	24	−27
Cingulum_Mid_R	209	−6.2417	0	−27	27

Abbreviations: CBF, cerebral blood flow; L, left; MNI, Montreal Neurological Institute; R, right; ReHo, regional homogeneity.

**TABLE 4:** Brain region with significant alteration of CBF/ALFF ratio by dobutamine infusions.

Regions	Cluster size (voxels)	Peak *t* values	MNI coordinates		
			*x*	*y*	*z*
Postcentral_L	12413	23.6258	48	24	−27
Frontal_Sup_Medial_R	342	7.8781	21	51	6

Abbreviations: ALFF, amplitude of low frequency fluctuations; CBF, cerebral blood flow; L, left; MNI, Montreal Neurological Institute; R, right.

### 3.5 Correlation Analysis Between Regional NVC and SBP

**FIGURE 5:**
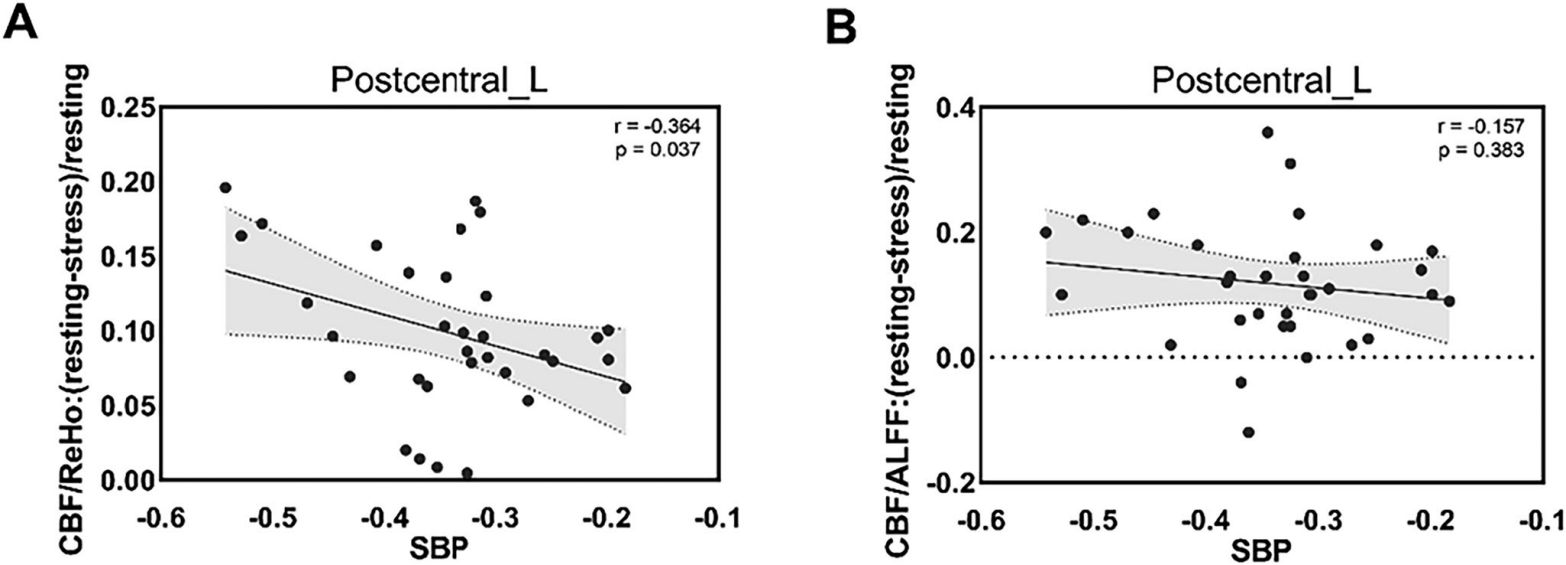
Correlation analysis between brain function and SBP in the left postcentral. (A) The percentage change of the CBF/ReHo ratio in the left postcentral brain region was negatively correlated with that of the SBP (r = –0.364; *p =* 0.037). (B) No correlation was found in the left postcentral brain region between CBF/ALFF and SBP. ALFF, amplitude of low‐frequency fluctuation; CBF, cerebral blood flow; L, left; ReHo, regional homogeneity; SBP, systolic blood pressure.

To investigate whether the alterations in the CBF/ReHo and CBF/ALFF ratio induced by acute stress were correlated with SBP, correlation analysis was performed between the alterations of CBF/ReHo ratios and CBF/ALFF ratios and the change of SBP. Under stress state, the percentage of change in the CBF/ReHo ratio in the left postcentral gyrus was significantly correlated with the change in SBP (Figure 5A; *r* = −0.364; *p =* 0.037; Mann–Whitney *U*‐test). However, there was no significant correlation in the left postcentral gyrus between the CBF/ALFF ratio and SBP (Figure 5B; r = −0.157, *p =* 0.383, Mann‐Whitney *U*‐test).

### 3.6 Predictors of Increase in NVC Coupling

We found that the CBF/ReHo ratios in the left postcentral brain region of the 38 participants increased from the resting state to the stress state. As shown in Table 5, binary logistic regression analysis revealed that DBP under the resting state (OR 0.817, 95% CI 0.692–0.965, *p =* 0.017) and SBP under the stress state (OR 1.218, 95% CI 1.067–1.390, *p =* 0.004) were independently associated with the increase in CBF/ReHo ratios in this region.

**TABLE 5:** Multivariate logistic regression analysis for predicting impact of dobutamine on NVC.

Variable	OR	95% CI	P
DBP in resting state	0.817	0.692∼0.965	0.017
SBP in stress state	1.218	1.067∼1.390	0.004

Abbreviations: CI, confidence interval; DBP, diastolic blood pressure; NVC, neurovascular coupling; OR, odds ratio. SBP, systolic blood pressure;

## 4 Discussion

The present study demonstrated there were NVC modulations under dobutamine‐induced acute stress, as evidenced by that dobutamine‐induced acute stress increased the NVC in brain regions of the anterior circulation, mainly in the left postcentral cortex, and that the increase in CBF/ReHo ratios was associated with DBP in the resting state and SBP in the stress state.

### 4.1 Increased NVC in Brain Regions of Anterior Circulation

This study showed that the CBF‐ReHo and CBF–ALFF coupling across voxels within the GM was significantly higher under the stress state than the resting state. These results suggested that acute stress played an important role in NVC in the whole brain, notably in the anterior circulation, given that the postcentral cortex and middle cingulate cortex are mainly supplied by the anterior circulation. This was consistent with a previous study (Jeffrey‐Gauthier et al. 2013) indicating that significantly higher NVC in rats during nociception processing was accompanied by higher systemic blood pressure but may be different in the stress nature, that is, stress induced by dobutamine in the present study and by electrical stimulation in the previous study (Jeffrey‐Gauthier et al. 2013). But in an in vivo mice study, NVC by using two‐photon imaging was found under acute stress induced by dobutamine, which was inconsistent with our study (Han et al. 2020). Furthermore, application of phenylephrine, a vasopressor similar to dobutamine and widely used in clinics, was found to be associated with a trend toward larger alternation of oxygenation in the frontal lobe of diabetic patients (Brassard et al. 2014). These results provide evidence for the proposal that the anterior circulation lobe is more vulnerable to the effects of acute stress.

Specifically at the brain region level, significantly higher CBF/ReHo ratio was found in the postcentral cortex and significantly lower CBF/ReHo ratio in the middle cingulate cortex under acute stress compared to the resting state. Neurophysiological studies described dedicated representations for body motion and somatosensation in the postcentral gyrus (Mastria et al. 2023; Iwamura et al. 2002). This enhanced NVC, on the one hand, may be due to the significant decrease of CBF in the postcentral cortex and, on the other hand, probably was caused by the passive tactile stimulation during the procedure of dobutamine injection. In the limbic system related to the production, processing, learning, and memory of emotions, the middle cingulate cortex is an essential component (Di Cesare et al. 2021; Domic‐Siede et al. 2021; Rolls et al. 2023). It is well established that acute stress has a major impact on memory at the cellular level through stress mediators, including catecholamines, such as adrenaline and noradrenaline (Schwabe et al. 2022). Consequently, this significant lower NVC in the middle cingulate cortex may be related to that dobutamine, predominantly a β1‐adrenergic agonist, could be the stress mediator. Recently, several studies suggested that metabolic activity in the amygdala and ventromedial prefrontal cortex served as a replacement for neural activity related to stress by using ^18^F‐FDG‐PET/CT, which could quantify the metabolic activity and enable simultaneous estimation of arterial inflammation (Mikail et al. 2024; Roy et al. 2012; Gangopadhyay et al. 2021). However, it is worth noting that the CBF/ReHo and CBF/ALFF ratios are indirect indicators of neurovascular coupling. Although they provide valuable insights into the balance between perfusion and neural activity, they do not directly capture the dynamic temporal relationship between neuronal firing and vascular response. Future studies using synchronous electrophysiological recordings or calibrated fMRI may provide more direct evidence.

Furthermore, there is no clear evidence supporting the laterality of NVC so far, and, to our best knowledge, our study was the first comparative analysis between bilateral and unilateral postcentral cortex and middle cingulate cortex, which showed the laterality of NVC under different states. This evidence was similar to the laterality of metabolic activity in the amygdala induced by stress (Mikail et al. 2024).

### 4.2 Predictors of Increase in Neurovascular Coupling

Our study suggested that NVC was associated with blood pressure parameters: DBP during the resting state and SBP during stress, indicating that blood pressure was a predictor of increase in NVC. This association highlighted the interplay of cerebrovascular and cardiovascular responses under stress and supported the notion that blood pressure fluctuations influence brain perfusion and neural synchrony (Macefield and Henderson 2016). Previous research has demonstrated that acute stress elevated both SBP and DBP, leading to changes in cerebral autoregulation and affecting perfusion dynamics (Tzeng et al. 2014). Our findings suggested that the CBF/ReHo ratios were sensitive to baseline DBP in the resting state, possibly reflecting underlying autonomic tone that influences cerebrovascular tone and stability. In contrast, during acute stress, elevated SBP may play a more direct role in modulating NVC, perhaps by exceeding autoregulatory thresholds in certain brain regions. This dynamic interaction between blood pressure and CBF/ReHo ratios provides insight into how acute stress may exacerbate cerebrovascular strain in individuals with pre‐existing cardiovascular conditions, supporting findings that heightened SBP responses to stress were linked with neurovascular dysregulation (Montano et al. 2009; Chu et al. 2024). The observed associations with blood pressure indicate that cardiovascular parameters could serve as accessible markers for predicting cerebrovascular responses under stress. Future research should focus on validating these findings in larger groups and investigating the influence of chronic stress on NVC to gain a deeper understanding of its long‐term effects on brain structure and cognitive function. Additionally, investigating whether similar NVC alterations occur with different types of stressors (e.g., psychological stress, traumatic stress, and environmental stress) could broaden our understanding of brain‐heart interactions across stress modalities.

Another physiological factor that could potentially influence our findings is the change in arterial carbon dioxide (CO_2_) during the dobutamine‑induced stress condition. End‑tidal CO_2_ was not directly measured in this study; therefore, the potential impact of CO_2_‑related cerebrovascular reactivity cannot be completely excluded. It is well known that alterations in arterial CO_2_ strongly modulate CBF through vasodilatory and vasoconstrictive mechanisms (Walsh et al. 2024; Alan et al. 2026). Although participants were instructed to maintain normal breathing throughout both resting and stress scans, and no symptoms of hyperventilation or breath‑holding were observed, small variations in respiration may still affect regional CBF. Consequently, part of the observed stress‑related CBF changes or neurovascular coupling alterations might reflect a physiological response secondary to ventilation effects rather than purely neural modulation.

### 4.3 Brain‐Heart Connections Under Acute Stress

The arterial‐brain circuit and the cardiac‐brain circuit are two main subcircuits that have been recently described in earlier research (Mohanta et al. 2023). This study indicated that the artery‐brain circuit appears to interact with the heart‐brain circuit even in healthy humans, which not only underscores the complexity of the brain‐heart axis but also hints at potential implications for various neurological and cardiovascular conditions (Liu et al. 2022)^.^ This study provided evidence for the intricate connections between brain and heart, given that acute stress induced by dobutamine acting directly on the heart had an effect on NVC, which was similar to earlier studies showing the association between higher regional CBF and posttraumatic stress disorder (Mikail et al. 2024; Bonne et al. 2003). However, another study indicated there was a decrease in CBF linked to acute posttraumatic stress disorder (Zhe et al. 2016). The discrepancy between these studies might be attributed to the differing time intervals for MRI imaging, with one study conducted three months apart and the other six months apart. A second interpretation for this discrepancy could be the disparity in imaging modality.

### 4.4 Limitations

There were several limitations in this study. First, assessment of cardiac function during dobutamine injection, which is essential to reveal the relationship between heart and brain, was not performed in the present study. Second, the female/male imbalance of participants in the study may limit the capability to explore the gender difference of dobutamine‐induced stress. Third, no vehicle such as saline was used as a control in the study to exclude the effect of tactile injection. Fourth, future investigations should incorporate simultaneous monitoring of respiratory parameters, including end‑tidal CO_2_, or use calibration paradigms that account for CO_2_‑induced cerebrovascular sensitivity. Such improvements would enable a more precise differentiation between neural and vascular components of stress‑induced NVC changes and further strengthen the physiological interpretability of this approach.

## 5 Conclusions

In summary, dobutamine‐induced acute stress modulated NVC dynamics, highlighting the sensitivity of cerebrovascular regulation to transient sympathetic activation. Our results further indicate that individual differences in baseline and stress‐related blood pressure are associated with the magnitude of NVC responses: participants with lower resting diastolic blood pressure (DBP) and higher systolic blood pressure (SBP) during acute stress were more likely to exhibit enhanced NVC. These findings suggest that peripheral cardiovascular reactivity may play a key role in shaping central neurovascular responses, providing novel insight into the mechanisms linking the brain and heart under acute stress conditions. Importantly, this work underscores the potential of NVC as a physiological biomarker for individual cerebrovascular adaptability, which could inform risk stratification and targeted interventions in both healthy individuals and populations susceptible to stress‐related cerebrovascular events.

## Author Contributions

P.R., P.Q., and Z.W. designed the experiments. P.R., X.M., L.C., T.Z., Y.L., and L.Z. conducted the experiments and completed the data collection. R.W., P.R., Y.L., T.Z., F.Y., and P.Q. analyzed the data. R.W. and P.R. wrote the initial draft. P.Q. and Z.W. supervised the entire experiment and revised the draft article. Z.W. acquired the financial support for the experiment. All authors have approved the final version of the manuscript and agree to be accountable for all aspects of the work. All persons designated as authors qualify for authorship, and all those who qualify for authorship are listed.

## Funding

This work was supported by the Space Medical Experiment Project of the China Manned Space Program (Nos. HYZHXM01012 and HYZHXMH01005), the Beijing Hospitals Authority Innovation Studio of Young Staff Funding Support (No.202302), and Beijing Scholar 2015.

## Ethics Statement

The experimental protocol was established according to the ethical guidelines of the Helsinki Declaration and was approved by the Beijing Friendship Hospital Research Ethics Committee (2020‐P2‐155).

Consent

Before the experiment, all participants signed informed consent forms.

## Conflicts of Interest

The authors declare no conflicts of interest.

## Data Availability

The data used to support the findings of this study are available from the corresponding authors upon request.
